# Transport experiences of people with disabilities during learnerships

**DOI:** 10.4102/ajod.v11i0.936

**Published:** 2022-10-18

**Authors:** Amanda E. Gibberd, Ntombizivumile Hankwebe

**Affiliations:** 1 Department of Transport, Pretoria, South Africa; 2 Avivah Occupations Therapists, Pretoria, South Africa

**Keywords:** universally accessible transport, students with disabilities, learnerships, SETAS, economic opportunity

## Abstract

Transport is a known national barrier for people with disabilities in South Africa. It is similarly identified as a barrier in learnerships and economic opportunity programmes. This article discusses the extent to which transport is a barrier during learnerships for students with disabilities. The Department of Transport administered an online evaluation questionnaire to a random sample of students with disabilities. Results were coded in terms of ‘barriers to access’ and ‘barriers to participation’. The data were organised into themes. The collated evidence is discussed in this article. The findings demonstrated that transport barriers were present in different modes of transport and different parts of the travel chain. However, the findings also demonstrated the negative impact of transport on the learnership experience and economic opportunities. The findings indicated that inaccessible transport is an integral cause of learnership incompletion for students with disabilities, where the universal accessibility of both transport and the built environment are a prerequisite need. Most students with disabilities reported that transport was not a barrier to learnership participation or that problems with transport could be resolved. Nevertheless, it was one of the identified barriers that negatively affected learnership participation experiences. It was a significant barrier to learnership completion for students with the most severe experience of disability. The sample consisted of only 32 students and a high number of unspecified responses. Evidence from other studies indicates that transport for all persons with disabilities remains a barrier warranting further examination, because public transport has remained inaccessible for over 23 years. Further research is required to verify this study and to investigate learnership cost–benefit for all students.

## Background

This article describes the impact of transport on students with disabilities participating in learnerships. Research on learnership experience identifies a range of barriers to access for students with disabilities participating in leanerships. Transport is identified as a known barrier (DoT 2020; Mahembe 2016; Mqikela 2015). Over the past 23 years, transport has also been identified as a barrier by people with disabilities who are not students (DoT 1999, 2020).

The Department of Transport (DoT) wished to explore how the barrier of transport affected students with disabilities who are participating in learnerships.

## Learnerships and transport in South Africa

The South African national learnership programme has developed over several decades. At the end of apartheid in 1996, apprenticeships provided artisanal skills administered by 33 training boards. These became 23 Sector Education Training Authorities or (SETAS) and were streamlined to 21 by 2016 (Department of Higher Education and Training [DHET] 2011). The SETAS introduced ‘learnerships’ as a new method of knowledge acquisition designed for a post-school environment. Learnerships were seen as a holistic skill development system for post-school students with low qualification levels (Davies & Farquharson 2004), rather than gaining an artisanal skill alone.

In explaining the role of the SETAS, the Department of Higher Education and Training (DHET 2020a) defines SETAS as ‘Skills Development Levy institutions that have a critical role to play in linking education and training institutions with the world of work (2020a:17)’. The SETAS collect skills levies from employers within a particular work sector, creating funds within the sector for relevant education and training. Funds are then made available to employers through these training bodies for sector-relevant skills development and to students in the form of discretionary grants and bursaries so that they can attend courses relevant to a particular career path within the sector (DHET 2020a). Employers of a certain size pay a percentage of their income for this process, as required by *The Skills Development Levies Act* (Department of Labour 1999) through the payroll tax. The aim of the SETAS, and therefore learnerships, is to deliver a national skills development programme that responds to industry needs (Department of Labour 1998). Since 2010, The Department of Higher Education has been responsible for SETA oversight.

The SETAS provide levels of qualification through the National Qualification Framework (NQF), resulting in the achievement of an ‘NQF level’ ranging from 1 to 10, with one being the lowest (South African Qualifications Authority [SAQA] 2012). The National Skills Development Plan (NSDP) guides skills programmes run by the SETAS through a National Skills Development Strategy (NSDS).

People with disabilities are under-represented in employment, and national targets on employment for people with disabilities have not been met (Department of Women, Youth, and People with Disabilities [DWYPWD] 2016). There are conflicting government views on the success of learnerships in achieving their aim of skills development and employment for people with disabilities. The National Development Plan or NDP (National Planning Commission [NPC] 2011) is South Africa’s national plan to overcome poverty, unemployment and inequality. The NDP identifies skills development as one of the three top priorities to grow jobs, capacity and a capable state (NPC 2011:27). It states that ‘Learnerships have facilitated entry to the labour market for unemployed people’ (NPC 2011:323).

Yet in 2011, the NSDS identified that the skills development element of learnerships for students with disabilities had failed (DHET 2011). Despite this, the 2020 post-school education and training analysis (PSET), a National Planning Commission document on skills development to 2030, does not address this failure (NPC 2020). Furthermore, the Department for Higher Education and Training report on skills supply and demand (DHET 2020b) fails to identify the problem at all. There is no mention of people with disabilities. They have simply now been omitted.

Transport, especially public transport, is an ongoing barrier for people with disabilities. Complaints have been laid with the DoT through the departmental complaints system (DoT 2020). Women have also laid complaints through the same system due to their experience of gender-based violence on public transport. Existing national studies on students with disabilities in learnerships (Mahembe 2016; Mqikela 2015) similarly demonstrate barriers in various parts of the transport travel chain.

Mqikela (2015) and Mahembe (2016) identified the following barriers. The proximity of transport to the workplace or training venue was a barrier for 30.0% of students with disabilities (Mahembe 2016). An additional 17.7% found access to buildings from public transport problematic, including a 30 min – 45 min walk to the destination (Mqikela 2015). This distance is simply too far for some students with disabilities (South African Bureau of Standards [SABS] 2011). Almost half (44.3%) found onsite external routes inaccessible within the learnership environment (Mahembe 2016). According to Mahembe (2016), if buildings or transport were inaccessible, students with disabilities were left out of meetings or training. Regardless of transport mode, both these studies show that students with disabilities leave home very early in the morning, use more than one mode to get to work on time and then get back home again, thus creating a longer working day than students without disabilities and a more expensive learnership experience (Mahembe 2016; Mqikela 2015).

The extent of the effect of these transport-related barriers on learnership completion is not well documented, due to a lack of integrated data in foundational learnership studies. For instance, foundational studies on learnerships include no biographical information on disability, but only gender and race (Kruss et al. 2014; Rankin, Roberts & Schöer 2014). As with Mqikela (2015) and Mahembe (2016), these two studies provide no clear link between learnership completion and access to work or economic opportunity. Whilst it is likely that the Rankin et al. (2014) and Kruss et al. (2014) studies covered students without disabilities alone, Mqikela (2015) and Mahembe (2016) categorically cover students with disabilities. The lack of a clear relationship between attending a learnership and accessing economic opportunities in both sets of studies signifies that learnerships may not achieve their stated aim. Despite this, funding made available for learnership programmes has risen dramatically over 20 years (National Treasury 2001, 2019).

The complaints received by the DoT between 2010 and 2020 from students enrolled for learnerships indicated that learners with disabilities were unable to complete learnerships due to transport barriers, thus supporting the findings in Mqikela (2015) and Mahembe (2016). Students with disabilities wished to lay complaints about these transport services with the DoT. However, the students who complained also indicated that they repeated learnerships because of incomplete qualifications and never entered employment. It was not clear from these complaints whether public transport was the only barrier to learnership completion and the lack of attainment of qualifications or if other barriers within the learnership also prevented learnership completion.

## Methodology

The DoT provided institutional permission to implement the authors’ questionnaire as part of the Department’s monitoring and evaluation mandate in 2020. The authors emailed evaluation questionnaires to a random sample of 55 learnership students with disabilities who used public transport to get to and from their learnerships. A response rate of 58% (32 students) was achieved. Whilst it is acknowledged that this sample is too small to be generalised, the response rate indicates a desire for the DoT to understand the situation of participating students.

The evaluation questionnaire focused on two topic areas: the recruitment of learnership candidates and workplace experience during the learnership. It covered the subject of transport in both areas, as well as other subject matter relating to the learnership experience. The questionnaire included open and closed questions to obtain both qualitative and quantitative information. It covered the following information-set categories: biographical data, qualifications, barriers to access and barriers to participation, with transport-related questions for both the recruitment and workplace experience phase. Reasonable accommodation, workplace modifications and learnership experience were included as separate categories. This article only reports on the transport-related responses.

The authors coded and categorised the results into themes emerging from the responses to the evaluation questionnaire and then analysed these themes using a rights-based assessment framework. Emergent themes (aside from transport in both the areas of learnership recruitment and workplace experience) were unresolved physical barriers to access, satisfaction with reasonable accommodation or in overcoming barriers and future employment concerns. Responses on transport were compared to other barriers to learnership completion.

### Limitations of the article

Evaluation questionnaire feedback is always limited, in that only those with complaints or concerns respond. This review is only based on the complaints to the DoT. Data from government sectors other than transport, such as that from corporate and health organisations, is not included. Employers, SETA staff and training providers were not consulted. Onsite audits or interviews were not conducted.

## Transport as a barrier throughout the learnership

**FIGURE 1:**
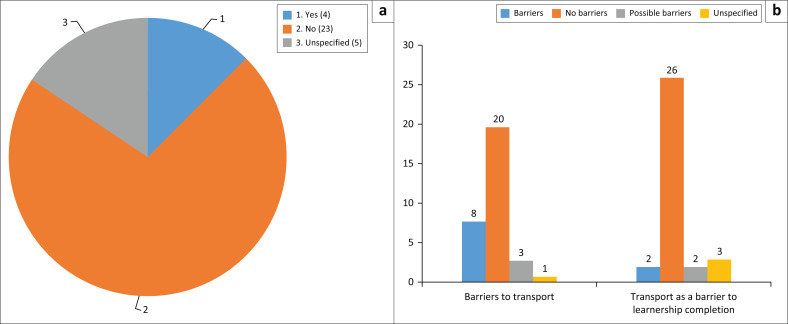
Barriers to transport during the learnership, both during recruitment and in the workplace. (a) Identification of transport barrier during recruitment, (b) Experience of a transport barrier during entire learnership.

Despite the limitations described, most students with disabilities did not identify transport as a barrier, as Figure 1 demonstrates.

**FIGURE 2:**
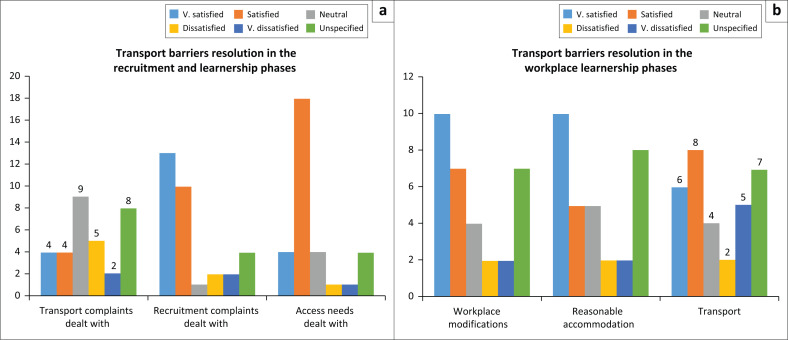
Workplace barrier resolution on transport or other universal access factors. (a) Recruitment: barrier resolution, (b) Workplace barrier resolution.

Whilst Figure 1 shows that students with disabilities in learnerships who are unable to use transport are small in number and are a minority group, around 75% of this same group identified significant problems worth discussing regarding transport during the recruitment and workplace phases, which are illustrated in Figure 2, through the resolution of complaints on transport and other barriers.

Of the 32 students, 24 registered transport complaints during the recruitment phase, which is 75%. Eight were satisfactorily resolved, and seven were unsatisfactorily resolved, which in both cases is about a third. A quarter of the sample cannot be accounted for due to the unspecified responses. During the workplace phase, 25 students (78%) registered transport complaints. Of those complaints registered, 14 were satisfactorily resolved and seven were unsatisfactorily resolved. This means that around one-half were satisfactorily resolved and a third were not. A fifth of the sample cannot be accounted for due to unspecified responses.

The neutral response is difficult to interpret. It could indicate satisfaction, or it could indicate resignation. If it indicates satisfaction, 70% of transport complaints were successfully resolved in the recruitment and workplace phases. If it indicates resignation, then 66% of complaints were unsatisfactorily resolved in the recruitment phase and 44% in the workplace phase. The high number of unspecified responses relative to the sample size means that the survey data is relatively incomplete and requires verification.

During the recruitment phase, Figure 2 shows that transport complaints were less satisfactorily dealt with than recruitment operations and access need-related complaints. In the workplace phase, modification and reasonable accommodation barrier resolution rates are higher than transport barrier resolution.

Nevertheless, it appears that most students with disabilities who experienced problems with transport were able to resolve them, although the extent of satisfactory resolution, including the neutral response, remains a concern to the DoT.

Nationally, there is both historic and current difficulty meeting employment targets for people with disabilities (DWYPWD 2016). This study showed that 84% of students with disabilities were likely to consider completing other learnerships, and half of these students would do so because of the unavailability of work. Only 40% of the students with disabilities in this study had qualifications above matric, which affects employability. Besides not having sufficient qualifications, 80% were taking a second or third learnership, and 99% were over 25 years old.

One possibility is that the neutral response in this study indicates that transport is not a significant problem for people with disabilities attending learnerships, based on the frequency of the complaints, and the indication that most transport problems can be resolved. If transport is a resolvable problem for most students with disabilities, and the findings of this survey hold in a larger survey, then most of the barriers to qualification completion for students with disabilities can be found in the education or learnership system, and not transport. The problem of employment, likewise, can be due to skills or labour market-related issues, and not transport.

Alternatively, the neutral response should be interpreted as negative. If this is the case, transport remains a significant barrier to learnership completion for students with disabilities. Whether or not the majority of students with disabilities experience an unresolved transport problem is immaterial in transport legislation. The severity of the problem remains the reason that complaints must be examined. The quotes below demonstrate that:

‘As an individual with a disability, the barrier that makes me miss good opportunities is always transport or accommodation. In most cases, we earn a stipend, not a salary, and the areas we get placed in are expensive. We cannot afford to pay for accommodation close to the workplace.’ (black; wheelchair user; Balfour)

‘Transport costs more than a stipend.’ (black, wheelchair user, Johannesburg)

‘Transport was the major problem that I had to deal with almost every day, and I nearly gave up on the learnership programme. Sometimes I would be late because taxi drivers don’t appreciate assisting someone using a wheelchair.’ (black; wheelchair user, East London)

‘Public transport is an issue, especially because I have a mobility disability. I find that after these learnerships, nothing is done for you; you go back home and remain unemployed.’ (black, wheelchair user, King William’s Town)

These survey quotes indicate the extent of multiple barriers to participation; the lack of accessible housing closer to areas of work where learnerships take place, the cost of transport for people with disabilities relative to income and the likelihood of unemployment on completion of the learnership. These quotes confirm DoT complaints received from other people with disabilities who are not students on learnerships (DoT 2020).

The qualitative responses to the open questions in the evaluation questionnaire covered the unwillingness of public transport operators to assist students with disabilities, inaccessible mini-bus taxis and insufficient income to afford on-demand services such as Uber, Bolt or metered taxis, which could be easier to use. The survey findings showed a relationship between the lack of access to transport resulting in absenteeism and the lack of punctuality at work, which led to learnership incompletion.

The Department of Women, Youth and Persons with Disabilities (Mqikela 2015) similarly concluded that the ‘barrier of transport’ was either caused by the distance of the destination from the origin, the distance of the transport stop to the venue, the inaccessibility of a particular transport mode or a combination of these factors (2015:25). The Mahembe (2016) study cites the cost of transport as a reason for learnership incompletion (2016:33), without clarifying whether increased transport costs are due to the travel distance or the inaccessibility of the design of transport vehicles, but concluding that the learnership income is nevertheless insufficient.

The quantitative information gathered during the authors’ survey supports the evidence in the qualitative data. For those negatively affected, regardless of the ‘stipend’ or income received from the learnership, public transport was either problematic or expensive or both. Although 41% received a monthly amount of over R3000 and 48% received around half this amount at R1500.00 or less, 75% experienced problems with transport, in both the recruitment and workplace phases, because of a physical or operational barrier that caused transport to be inaccessible and because the length of the transport journey meant that it was too expensive to afford.

The findings in Figures 1 and 2 from the authors’ survey show that where transport is a problem to access, it can be resolved for most students on learnerships. Nevertheless, these figures also show that doubling the amount of money that students receive is not sufficient to resolve transport barriers. The quotes from the qualitative data in the authors’ survey illustrate that the distance between where people live and their destinations creates a barrier caused by the sheer cost of transport relative to income, aside from the inaccessibility of a particular transport mode.

Furthermore, all of these factors inflate the cost of living for a student with a transport disability and decrease net income. Multiple barriers to participation are evident. If both transport and learnerships were accessible, barriers in transport and urban planning as related to housing will still prevent some people with disabilities from completing their learnership.

### Implications on the learnership experience

The authors’ evaluation questionnaire showed that a third of the students with disabilities had unresolved transport problems. Although this number does not constitute the majority, it is especially notable because of the inability of most employers to meet national employment and skills development targets for people with disabilities (DWYPWD 2016). Between two and four students were unable to complete their learnership because of a transport problem. This is also important because these are students with significant experiences of disability. Their inability to gain employment because of public transport inaccessibility remains a likely outcome.

The ‘barrier of transport’ was identified in Moving South Africa (DoT 1999) for people with disabilities and other categories of passengers with identified access needs. The authors’ findings support the conclusion from published research (Mahembe 2016; Mqikela 2015) as well as findings in the DoT complaints system (DoT 2020) that insufficient progress on universally accessible transport has been made since 1999. Over 23 years, public transport remains inaccessible to everyone.

There is a lack of acknowledgement of students with disabilities in recent nationally issued reports on learnership experience (DHET 2020b; NPC 2020). The authors wondered if any learnership barriers experienced by students with disabilities are acknowledged in all learnership research projects, both in terms of reference issued for these studies, and in learnership evaluation programmes. The lack of acknowledgement of people with disabilities in published reports creates a gap in the evaluation of transport as a barrier. It is not clear if by removing the barrier to transport and providing universally accessible transport, learnership completion and economic opportunity for people with disabilities will be achieved. If barriers within learnerships remain, universally accessible transport will achieve very little.

The DoT began to address inaccessible transport as a legacy project of the 2010 World Cup^TM^ (DoT 2009). The identified Integrated Public Transport Network (IPTN) municipalities have received a special allocation of between 5 and 6 billion rands annually, from 2010 to 2020, through a dedicated conditional grant (National Treasury 2010–2020). This has resulted in accessible transport systems in only six out of 13 IPTNS, covering only a fraction of each municipality. Complaints received on new municipal public transport systems continue to highlight significant problems with their inaccessibility. Currently there is no national programme to upgrade existing services, although universally accessible planning for transport is already legally required as a minimum standard (DoT 2016). Without a national change in the approach to universal design in both transport and urban planning as well as transport service operations, barriers to transport will likely remain (Gibberd 2021).

### Implications beyond the learnership experience

Despite the low post-learnership employment levels described in existing studies, students with disabilities continue to believe that post-learnership employment is attainable. The authors’ data support this finding; most students who participated in the survey (about 75%) attend learnerships to achieve employment.

The authors’ survey shows a concern from students about future barriers to work aside from transport. These barriers include inaccessible built environments and inaccessible workplace information. The students’ concerns were that these barriers were not being addressed.

With 77% of the survey participants indicating a concern that future barriers to work will not be dealt with, most students with disabilities believe that their access needs will not be identified and that reasonable accommodation will not be implemented. Their future beliefs relate to their current experience. In the authors’ study, 94% of students identified an access need in either the recruitment or the workplace phase of the learnership. Figure 2 shows that in transport, at least 40% of those barriers remained unresolved. Other results showed that 37.5% of students were less than satisfied with the reasonable accommodation measures made.

## Conclusion

The results of the authors’ evaluation questionnaire found that inaccessible transport is a significant barrier to learnership completion, especially for students with the most severe experience of disability. Transport was found to be a barrier due to its inaccessible planning, design, operation and cost of transport journeys. The lack of access to transport appeared to undermine students with disabilities, leading to unpleasant and demoralising learnership experiences or learnership incompletion. Transport as a barrier to access for people with disabilities and others with universal access needs was identified in early research over two decades ago and remains largely unaddressed (DoT 1999, 2020).

Secondly, the results of the authors’ evaluation questionnaire indicate other post-learnership employment concerns amongst people with disabilities, aside from transport. This finding is particularly problematic, principally for the DoT. Transport is a barrier for students with disabilities. However, if transport became universally accessible, the ‘disability of unemployment’ remains. It is interesting that foundational studies on learnerships do not identify ‘disability’ in biographical information (Kruss et al. 2014; Rankin et al. 2014) or the extensive barriers that people with disabilities face. Disability studies are separately available; nevertheless, people with disabilities have not been included in the mainstream as national legislation requires, neither in transport nor in studies on learnership experience. Also in need of reform, are the current learnership and SETA structures; which appear not to bridge the post-school employment gap as the National Development Plan claims, particularly for people with disabilities but also for those without, and to not achieve it at a substantial cost.
